# Fluorescence Spectroscopy Study of Protoporphyrin IX in Optical Tissue Simulating Liquid Phantoms

**DOI:** 10.3390/ma13092105

**Published:** 2020-05-02

**Authors:** Huihui Lu, Francesco Floris, Marc Rensing, Stefan Andersson-Engels

**Affiliations:** 1 Biophotonics @ Tyndall, IPIC, Tyndall National Institute, University College Cork, T12 R5CP Cork, Ireland; stefan.andersson-engels@tyndall.ie; 2 Photonics Packaging Group, IPIC, Tyndall National Institute, University College Cork, T12 R5CP Cork, Ireland; francesco.floris@tyndall.ie (F.F.); marc.rensing@tyndall.ie (M.R.); 3 Department of Physics, University College Cork, T12 K8AF Cork, Ireland

**Keywords:** protoporphyrin IX, optical phantoms, fluorescence spectroscopy, optical properties, tissue diagnostics

## Abstract

Fluorescence spectroscopy has been extensively investigated for disease diagnosis. In this framework, optical tissue phantoms are widely used for validating the biomedical device system in a laboratory environment outside of clinical procedures. Moreover, it is fundamental to consider that there are several scattering components and chromophores inside biological tissues and the interplay between scattering and absorption may result in a distortion of the emitted fluorescent signal. In this work, the photophysical behaviour of a set of liquid, tissue-like phantoms containing different compositions was analysed: phosphate buffer saline (PBS) was used as the background medium, low fat milk as a scatterer, Indian ink as an absorber and protoporphyrin IX (PpIX) dissolved in dimethyl formamide (DMF) as a fluorophore. We examined the collected data in terms of the impact of surfactant Tween-20 on the background medium, scattering effects and combination of scattering and absorption within a luminescent body on PpIX. The results indicated that the intrinsic emission peaks are red shifted by the scattering particles or surfactant, whilst the scattering agent and the absorbent can alter the emission intensity substantially. We corroborated that phantoms containing higher surfactant content (>0.5% Tween 20) are essential to prepare stable aqueous phantoms.

## 1. Introduction

Fluorescence spectroscopy is seen as a promising diagnostic tool thanks to its inherent rapid response, combined with high sensitivity and specificity rate. This makes it an ideal method for disease diagnosis in the fields of biological and biomedical applications, specifically for tissue diseases. When tissues are illuminated with ultra-violet or visible light, biological molecules can absorb the energy carried by the impinging light and re-emit it at a longer wavelength. This can be used to capture the features of endogenous or exogenous fluorophores in the form of an injectable fluorescent molecule to differentiate tumour and normal tissues in a variety of organ systems [1,2,3]. Most common endogenous tissue fluorophores include tryptophan, tyrosine, phenylalanine, collagen, elastin, reduced nicotinamide adenine dinucleotide (NADH), flavin adenine dinucleotide (FAD) and porphyrins [4]. Each of these molecules has unique excitation and emission spectra. There are also various scattering components and chromophores in biological tissues. Photons incident on a tissue undergo substantial scattering and absorption when emerging from the surface. The interplay of absorption and scattering can result in distortion of the fluorescent signal from tissues [5,6]. Disentangling effects of scattering and absorption have already been investigated. Müller et al. [7] developed a model based on photon-migration theory and information from simultaneously acquired fluorescence and reflectance spectra, in order to extract the intrinsic fluorescence from turbid media. Wu et al. [8] used a non-negative matrix factorization algorithm to generate a fluorescence excitation emission matrix that corresponded to the fluorophores in biological tissue, including tryptophan, collagen, elastin, NADH and FAD.

Protoporphyrin IX (PpIX), a heterocyclic organic compound consisting of four pyrrole rings, is the final product in the heme biosynthetic pathway [9]. In the field of neurosurgery, administration of an exogenous fluorophores or a fluorophores precursor, such as 5-aminolevulonic acid (5-ALA), is used to help guide surgery [1,4,10]. 5-ALA is taken up by glioma cells where a breakdown of the blood-brain barrier (BBB) has occurred, but not in normal brain tissue. This permits fluorescence discrimination of the tumour and normal tissue. Researchers in this field have developed a variety of approaches to quantify PpIX fluorescence emission from tissue [11,12]. Optical tissue phantoms are widely used for validating prototype medical device systems in laboratory environments outside of clinical procedures.

In this framework, our goal is to offer an additional procedure for optical fluorescent tissue phantoms in order to validate an integrated optical probe system for tissue characterization, based upon diffuse reflectance and fluorescence.

With this in mind, the aim of this paper is to find and study a set of liquid, tissue-like phantoms with different compositions. We used PBS as the background medium, low fat milk as a scatterer, Indian ink as an absorber and PpIX as a fluorophore. PpIX can be dissolved in many organic solvents (e.g., dimethyl sulfoxide (DMSO), DMF, etc.), but it is very difficult to dissolve in water, and we are interested in water-based background media. Surfactants such as Tween are commonly added to aqueous PpIX phantoms to prevent aggregation [11,12,13,14]. They have an effect on the fluorescence spectra [13,15,16,17]; therefore, the role of Tween on background medium was also investigated.

## 2. Materials and Methods

### 2.1. Instrumentation

PpIX has a fluorescence emission property due to π-electron delocalization over the conjugated double-bond system. PpIX can be excited in different spectral regions, each offering advantages and disadvantages [13,18]. Commonly, blue light is used to excite the PpIX in fluorescence-guided surgery [12,19]. In this spectral region, blue light generates efficient fluorescence, however, the light is highly absorbed by haemoglobin. Excitement in the red wavelength yields a less efficient fluorescent signal, but avoids light attenuation by the blood and achieves deeper sampling. Using red light excitation has been applied for PpIX concentration monitoring during photodynamic therapy [20].

In this study, light at a wavelength of 415 nm was chosen for excitation. During the measurements, excitation light from a Fiber-Coupled LED (Thorlabs M415F3, Output power ~21.3 mW, FWHM 14 nm, Spot size ~1.3 mm) was projected on the samples and fluorescent emissions were collected using a QEPro spectrometer from Ocean Optics (Duiven, Netherlands), with a detection range from 400 to 1000 nm. Measurements were taken at an angle perpendicular to the excitation light [21]. Liquid phantom samples were placed in transparent plastics cuvettes with a 10 mm path length. To minimise inner-filter effects from the background medium, the background media without dye were recorded prior to each fluorescence measurement and subtracted from the raw data.

### 2.2. Liquid Phantom Samples Preparation

Tween-20, PBS, PpIX powder, and DMF were purchased from Merck (Wicklow, Ireland). Milk (Dawn^®^ low fat milk, produced in Kerry, Ireland) and Indian ink (Winsor and Newton^®^ black India ink, Cork, Ireland) were used as scatterer and absorber.

PpIX powder was dissolved in DMF to prepare PpIX stock solution as 10 mg/mL. Dilutions from this stock into PBS solution were used to achieve the desired PpIX concentration. A series of fluorescence emission spectra of PpIX were measured for different concentrations of PpIX.

In order to investigate the role of surfactant in background medium, 100 µL of PpIX (10 mg/mL) stock solution were diluted in 9.9 ml PBS solution containing different amount of Tween 20 (10%, 5%, 2.5%, 1.25%, 0.5%, 0.05%, 0.005%, 0.0005%, and 0%).

The selection of the optimal scatterers and absorbers in tissue phantoms strongly depended on specific applications [14,22,23,24]. In this paper, milk and Indian ink were introduced into PpIX solutions to investigate the influence of the scattering and the absorption on PpIX fluorescence emission spectra. One set contained different concentrations of milk without the background absorber. Another two sets contained both milk and Indian ink as a background absorber, and milk was used at two concentrations to simulate low and high scattering coefficients (130 and 260 cm^−1^). Indian ink was selected at five concentrations to simulate different absorption level coefficients (0.01, 0.29, 0.56, 1.11 and 2.21 cm^−1^). The properties of the scattering coefficient of milk and the absorption coefficient of Indian ink were determined by combination of the diffuse reflectance and transmittance spectroscopy measurement setup described in our previous work, [25,26] and were chosen within a range typical for biological tissue.

## 3. Results and Discussion

### 3.1. Effect of Surfactant on PpIX Fluorescence in Non-Turbid Phantom

**Figure 1:**
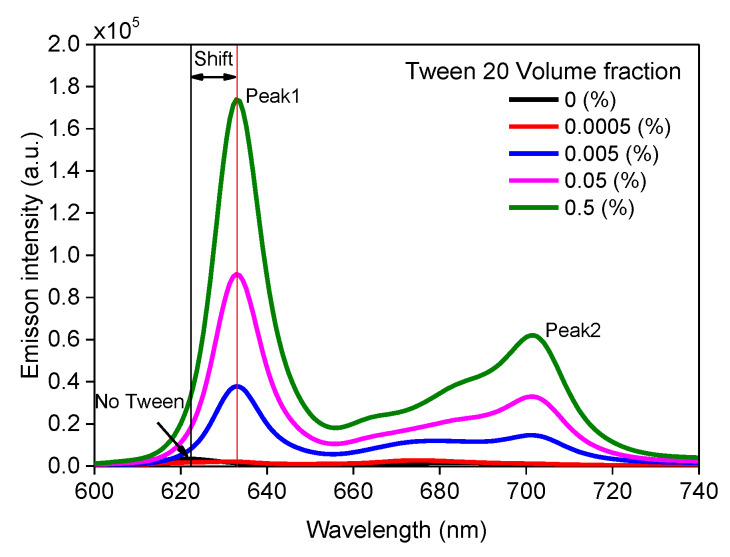
Fluorescence spectra (415 nm excitation) of PpIX in PBS buffer with different Tween 20 volume fraction.

The influence of Tween 20 on fluorescence spectra was investigated by diluting the same volume of PpIX stock solution in PBS buffer containing different volume fractions of Tween 20 (0.5%, 0.05%, 0.005%, 0.0005%, and 0%). Emission spectra are shown in Figure 1. It is observed that PpIX emission spectrum had two distinctive peaks at wavelengths of 622 nm and 684 nm, with the sample containing no surfactants. Emission peaks at 630 nm from PpIX solutions containing surfactant were red shifted (~10 nm). In addition, the intensity of the entire band significantly expanded.

**Figure 2:**
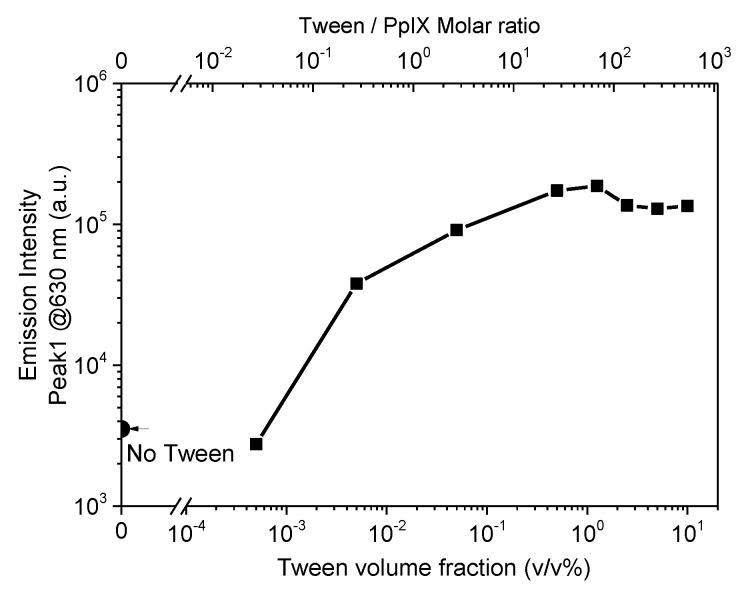
PpIX fluorescence peak intensity at 630 nm as a function of Tween 20 volume fraction (bottom x-axis) and Tween/PpIX molar ratio (top x-axis).

To further investigate the effect of Tween 20 on PpIX fluorescence, PpIX solution in PBS buffer containing higher volume fractions of Tween 20 (10%, 5%, 2.5%, 1.25%, 0.5%, 0.05%, 0.005%, 0.0005%, and 0%) were prepared, and the luminescence study was performed with these solutions. The emission peaks at 630 nm were plotted as a function of Tween 20 volume fraction and Tween/PpIX molar ratio in Figure 2. The emission intensities were observed to increase proportionally with the Tween volume fraction between 0.0005% and 1.25%. The PpIX solution containing Tween showed a strongly enhanced emission signal, with an approximate 49-fold increase at a Tween volume fraction of 0.5%, compared with that containing no surfactant. The emission intensities increase by 91% and 98% of the maximum signal in the sample containing 0.5% and 1.25% Tween respectively, compared with that containing no surfactant. Our experimental results agree with previous studies from Marois et al. [13], who showed a PpIX sample containing no surfactant with a reduction of 98% fluorescence emission, compared with that containing surfactant. The intensity of molecular fluorescence is greatly influenced by the solvent effect [27,28] and other medium variables, such as pH [28,29]. The purpose of our phantom was to simulate biological tissue, therefore PBS buffer with a pH kept at 7 was used as a background medium throughout the study. The enhancement of the fluorescence emission may be due to (1) the non-ionic surfactant increasing the solubility of hydrophobic PpIX; (2) at higher concentrations, micelles start to form and PpIX structured in micelles have different emission intensities. For Tween volume fraction in the range of 0.5% to 1.25%, the emission intensity exhibited a variation of only 7% between two concentrations. For Tween volume fraction in the range of 1.25% to 10%, emission intensity plateaued and slightly decreased. Tween 20 volume fraction (0.5% *v*/*v*) was therefore employed for further studies.

**Figure 3:**
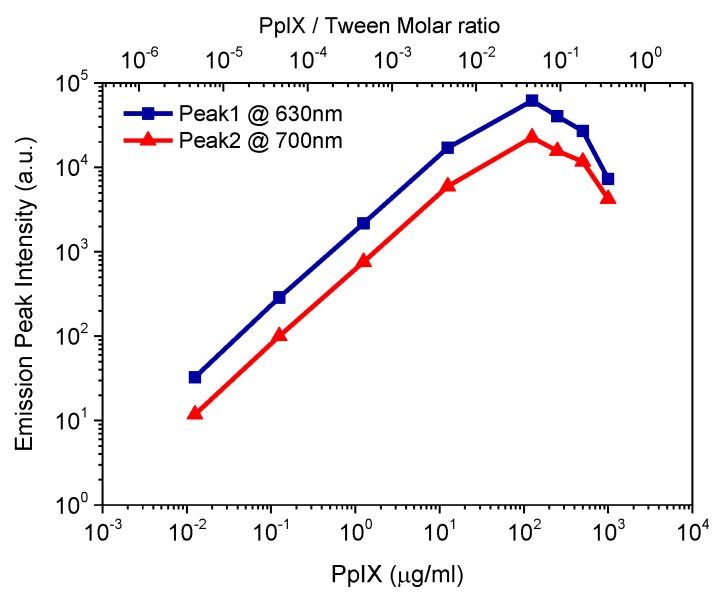
Emission peak intensity as a function of concentrations of PpIX in PBS solutions containing 0.5% (*v*/*v*) Tween 20 (bottom x-axis) and PpIX/Tween molar ratio (top x-axis).

Further studies were carried out by measuring a series of fluorescence emission spectra for different concentrations of PpIX in PBS solutions containing 0.5% (*v*/*v*) Tween 20. Emission peak intensity as a function of PpIX concentration is plotted in Figure 3. It reveals a maximum intensity around 125 µg/mL (PpIX/Tween molar ratio is 0.046) and shows that the emission signal decreased when going to higher PpIX concentration. PpIX has a fluorescence emission property due to π-electron delocalization over the conjugated double-bond system. Porphyrin presents high aggregates in a pH ranging from 3 to 7 [15,16], and aggregation through the occurrence of π-π stacking interactions. Figure 2 shows the molar ratio of Tween/PpIX to reach equilibrium was around 27 (at this point Tween 20 volume fraction is 0.5%, PpIX concentration is 100 µg/mL). Fluorescence emission intensity was expected to decrease due to the PpIX samples beginning to aggregate at higher concentrations. For further quantitative studies, PpIX concentration below 125 µg/mL was selected because a monotonic relationship between signal and dye concentration is preferred.

### 3.2. Effect of Scattering on PpIX Fluorescence

**Figure 4:**
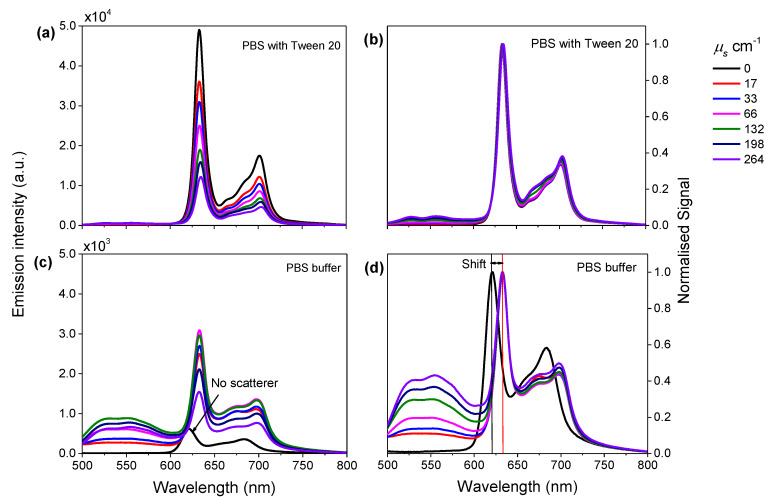
Fluorescence spectra (415 nm excitation) of PpIX with different concentrations of scattering agent and their normalised spectra (normalised to each spectrum peak intensity); in PBS buffer containing Tween 20 (0.5% *v*/*v*) (**a**,**b**) and PBS buffer containing no surfactant (**c**,**d**).

To investigate the impact of scattering on the intensity and the spectral shape of a PpIX fluorescence spectrum, low fat milk was used as the scatterer and was introduced into the aqueous phantom. The phantoms contained 100 µg/mL PpIX in PBS buffer containing 0.5% (*v*/*v*) Tween 20 with milk between 0% and 80% (*v*/*v*), corresponding to the scattering coefficient range, *µ*_s_, from 0 cm^−1^ to 264 cm^−1^ at a wavelength of 415 nm. The measured fluorescence spectra are shown in Figure 4a,b, showing that the normalised spectra shape (normalised to their peak heights) were all very similar to the spectrum with no scatterer added, and had two distinctive peaks at wavelengths of 632 nm and 701 nm. This observation showed that PpIX fluorescence was not red-shifted by the presence of the scatterer. This seemed not to be intuitive in terms of previous work from Ahmed et al. [5], which showed red shifts of the emission spectra peaks in the presence of a random scatter in luminescent bodies, with respect to the intrinsic fluorescence emission. The three main components of milk, besides water, are lipids, protein and lactose. The most abundant membrane lipids are the phospholipids [30]. Phospholipids have amphipathic properties, and therefore we hypothesised that this component in the milk may act as a surfactant. Similar experiments were further carried out in the PBS buffer containing no surfactant. Figure 4c,d show the red shifts in the emission spectra in the presence of the scatterer, with emission peak wavelength at ~630 nm and ~700 nm. These results are very similar to what we have observed in the previous section (Section 3.1 and Figure 1) on fluorescence spectrum of PpIX in PBS buffer with different Tween 20 volume fraction. This confirmed that milk in the phantom may act as a surfactant in the aqueous tissue-like phantom.

**Figure 5:**
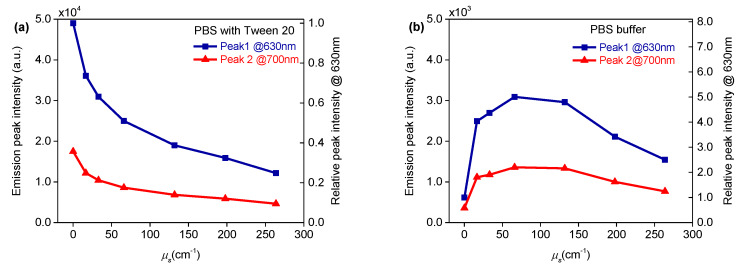
PpIX fluorescence peak intensity at ~630 nm (blue line) and ~700 nm (red line) plotted as a function of scattering coefficient (left y-axis). Relative emission intensities at 630 nm of PpIX samples containing different concentrations of scatterer, compared with that containing no scatterer as a function of scattering coefficient (right y-axis). (**a**) in PBS buffer containing Tween 20 and (**b**) PBS buffer containing no surfactant.

PpIX fluorescence peak intensities at ~630 nm and ~700 nm were plotted as a function of the scattering coefficient as reported in Figure 5 with left y-axis. Relative emission intensities at 630 nm of the PpIX sample containing different amounts of scatterer, compared with that containing no scatterer, were plotted as a function of scattering coefficient, which are shown in Figure 5 with right y-axis. In the case of the background medium as PBS buffer containing Tween 20 (Figure 5a), the fluorescence intensity decreased nearly exponentially, with increased scattering coefficient. The PpIX sample containing the scatterer at the scattering coefficient *µ*_s_ at 130 cm^−1^ (*µ*_s_’ = 13 cm^−1^ given anisotropy factor g = 0.9) reduced 64% of fluorescence emission, compared with that containing no scatterer. In the case of PBS buffer containing no surfactant, Figure 5b shows the fluorescence intensity increased with increased scattering coefficient at lower values of *µ*_s_, from 0 cm^−1^ to 66 cm^−1^. It is shown that the PpIX sample containing the scatterer at scattering coefficient *µ*_s_ at 66 cm^−1^ had an increase of 400% fluorescence emission compared with that containing no scatterer. For larger values of *µ*_s_, from 66 cm^−1^ to 264 cm^−1^, the fluorescence intensity decreased with increased scattering coefficient after reaching the peak value. The normalised spectra in Figure 4d indicate an increased absorption due to light scattering. In this regime, light undergoes a longer random-walk path and experiences more absorption, hence the decreases in emission intensity.

### 3.3. Effect of Combined Scattering and Absorption on PpIX Fluorescence

To investigate absorption effects on PpIX fluorescence in the presence of a scatterer, we conducted experiments using another two sets of phantoms containing milk and Indian ink as a background absorber. Milk was used at two concentrations to simulate low and high scattering coefficient (130 and 260 cm^−1^). Indian ink was selected at five concentrations to simulate different absorption coefficient levels (0.01, 0.29, 0.56, 1.11 and 2.21 cm^−1^).

**Figure 6:**
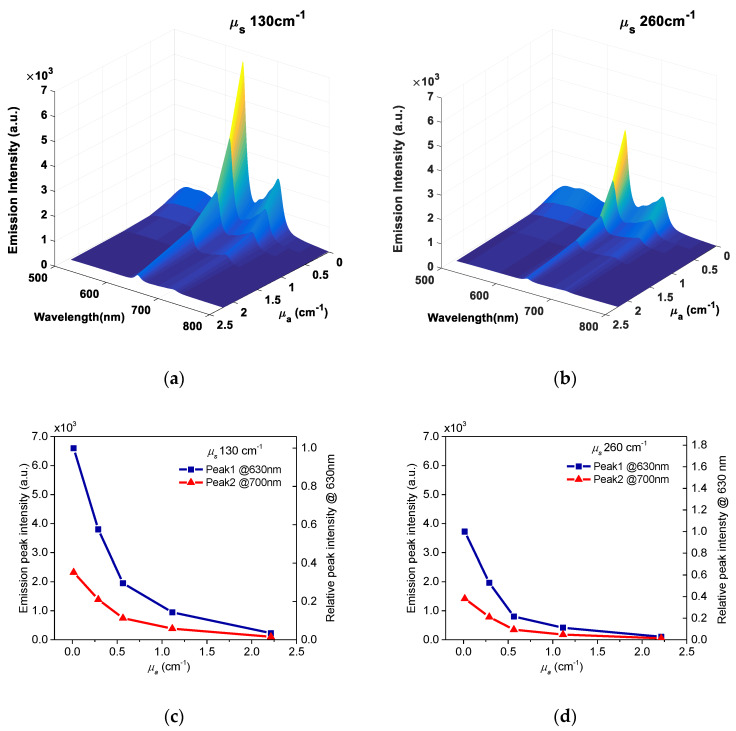
Fluorescence spectra (415 nm excitation) of PpIX in PBS solution as a function of wavelength at lower (**a**) and higher (**b**) scattering coefficients. Emission intensities as a function of absorption coefficient at lower (**c**) and higher (**d**) scattering coefficients. Relative emission intensities at 630 nm of PpIX sample containing different concentrations of absorber compared with that containing no absorber is shown in right y-axis.

Figure 6 shows an overview via a 3D diagram of the fluorescence spectra of PpIX in PBS solutions, as a function of wavelength and absorption coefficients at the level of *µ*_s_ equal to 130 cm^−1^ and 260 cm^−1^. To illustrate more clearly the absorption effects, PpIX emission peak intensity at ~630 nm and ~700 nm, plotted as a function of absorption coefficient, are also displayed in Figure 6c,d with the left y-axis. Relative emission intensities at 630 nm of the PpIX sample containing different concentrations of absorber, compared with that containing no absorber, were plotted as a function of absorption coefficient, which are shown in Figure 6c,d with the right y-axis. With increased absorption coefficient, the fluorescence intensity decreased. PpIX samples containing the absorber at the absorption coefficient *µ*_a_ at 1.1 cm^−1^ and 2.2 cm^−1^, showed reductions by 88% and 97% fluorescence emission, respectively, compared with that containing no absorber. Marois et al. [13] used whole blood as a background absorber and identified that there is an interaction between erythrocytes in whole blood and Tween, which can complicate the stability of PpIX fluorescence. In our experiments, we did not observe any decrease in the fluorescence signal over eight hours in freshly prepared samples in PBS solution containing Tween 20. Using whole blood as an absorber in phantoms can provide realistic tissue spectra and oxygenation function, while ink provided nearly flat absorption spectra. The selection of the optimal absorber in tissue phantoms strongly depends on specific applications [14].

### 3.4. Temporal Stability Study of PpIX Fluorescence

**Figure 7:**
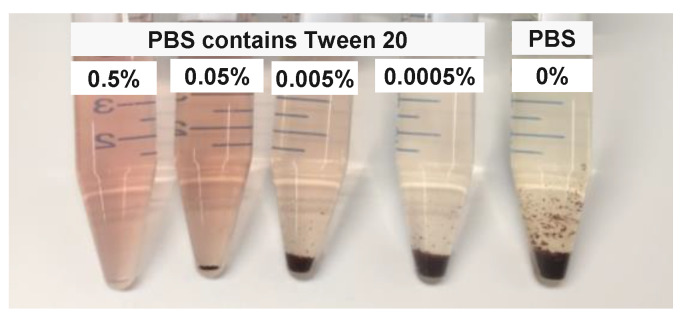
PpIX aqueous samples prepared in PBS containing different volume fractions of Tween 20 at day seven after preparation.

We investigated phantom stability in terms of aggregation, by comparing PpIX prepared in PBS buffer containing different concentration of Tween 20 (0.5%, 0.05%, 0.005%, 0.0005%, and 0%) stored at room temperature (20 °C). It is clearly shown in Figure 7 that there was no precipitation observed in PBS containing 0.5% (*v*/*v*) Tween 20 at day seven after samples were freshly prepared.

**Figure 8:**
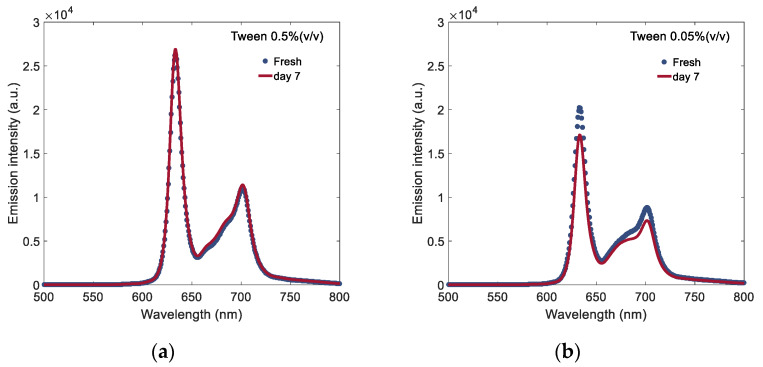
Fluorescence spectra (415 nm excitation) of PpIX measured at freshly prepared and at seven days after preparation in PBS buffer containing (**a**) Tween 20 (0.5% *v*/*v*) and (**b**) Tween 20 (0.05% *v*/*v*).

To further characterise the temporal stability of PpIX fluorescence signal in PBS containing surfactant, the sample was measured when it was freshly prepared and seven days after preparation. The measured fluorescence spectra of PpIX prepared in PBS containing two different concentrations of Tween 20 (0.5%, 0.05%) are shown in Figure 8. In PBS containing 0.5% (*v*/*v*) Tween 20 medium, the spectra of PpIX aqueous sample measured at day seven after preparation was nearly identical to that of the freshly prepared sample. Its spectra showed good stability in terms of the fluorescence signal intensity, while in PBS containing a lower concentration of Tween 20 (0.05% *v*/*v*), the fluorescence signal decreased at day seven after preparation.

## 4. Conclusions

In this paper, we presented the experimental results of surfactant Tween 20 volume fraction changes on background medium, scattering and combination of scattering, and absorption within a luminescent body on PpIX fluorescence, observed at the surface of the phantom samples. The data indicated that milk and Indian ink can be reliably used as scatterer and absorber in PpIX fluorescent phantoms. It is shown that red shifts occur in the emission spectra in relation to the intrinsic fluorescence emission in the presence of the scatterer and surfactant, whilst the scatterer and absorber can alter the emission intensity substantially. Phospholipids in the milk in the phantom act as a surfactant in the phantoms. The PpIX solution containing 0.5% (*v*/*v*) of Tween 20 presents a significantly enhanced emission signal compared with that containing no surfactant. We also corroborated that the surfactant content (>0.5% Tween 20) was essential to prepare stable aqueous PpIX fluorescent phantoms.
